# Advancing the Lusaka Agenda: the Global Financing Facility’s missed opportunities for catalysing sustainable health investment

**DOI:** 10.1080/16549716.2025.2555052

**Published:** 2025-09-09

**Authors:** Anne Musuva, Marie-Jeanne Offosse, Aloysius Ssennyonjo

**Affiliations:** a ThinkWell, Kenya Country Office, Nairobi, Kenya; b ThinkWell, Burkina Faso Country Office, Ouagadougou, Burkina Faso; c School of Public Health, Makerere University, Kampala, Uganda

**Keywords:** Global Financing Facility for Women, Children and Adolescents: Examining National Priorities, Processes and Investments, Global Financing Facility, Lusaka agenda, policy analysis, domestic resource mobilization, content analysis

## Abstract

This Commentary is part of the *Global Health Action* Special Issue titled *Global Financing Facility for Women, Children and Adolescents: Examining National Priorities, Processes and Investments*. The Issue examines the Global Financing Facility (GFF) through the lens of nine papers that explore the content and development processes of GFF country documents. While the GFF achieved technical alignment with national reproductive, maternal, newborn, child, and adolescent health priorities, it did not consistently translate into the mobilization of increased domestic resources. Loan-heavy financing structures substituted, rather than supplemented, public spending and intensified fiscal pressure in debt-constrained contexts. The expansion of results-based financing models has brought additional sustainability and equity concerns, with many initiatives collapsing post-project due to inadequate alignment with public finance systems. This falls short of the Lusaka Agenda’s strategic shift towards country-led sustainable financing. Stakeholder engagement, particularly in civil society organizations, is often late, limited, or superficial. The neglect of stillbirth and respectful maternity care in the GFF documents calls for a critical look at high impact underprioritized areas when making future GFF investment decisions. As global health aid retracts and low- and middle-income countries navigate debt pressures, global health initiatives must base investments in disease burden data and engage stakeholders meaningfully. To realize the Lusaka Agenda’s vision, the GFF must align with national public finance systems and secure sustainability beyond donor cycles.

## Paper Context

- **Main findings**: While Global Financing Facility country documents are generally aligned with country health priorities, they fall short of advancing the Lusaka agenda’s goal of country-led sustainable financing.
- **Added knowledge:** The Global Financing Facility has missed opportunities to mobilize domestic resources, design sustainable funding models, engage stakeholders meaningfully, and prioritize neglected but high-impact areas.
- **Global health impact for policy and action**: The Global Financing Facility’s investment decisions must be guided by disease burden data, aligned with sustainable financing, and informed by meaningful stakeholder engagement

## Background

This Commentary provides a sobering reflection on how global financing initiatives, while well-intentioned, often fall short of delivering systemic change as envisioned in the Lusaka Agenda. Launched in 2023, the Lusaka Agenda outlines a bold vision for the next generation of global health initiatives (GHIs) to become country-led, equitable, and sustainable [1]. It calls for five key shifts: 1) strengthening primary health care (PHC), 2) catalysing sustainable, domestically financed health services, 3) strengthening equity in health outcomes through joint approaches, 4) achieving strategic and operational coherence, and 5) coordinating approaches to products, research and development, and regional manufacturing. In light of the Lusaka agenda, we draw on findings from the *Global Health Action* Special Issue titled *The Global Financing Facility for Women, Children, and Adolescents: Examining National Priorities, Processes, and Investments* [2] together with our own experiences with the Global Financing Facility (GFF), to offer recommendations for guiding the future evolution of the GFF and other GHIs.

The sudden global health funding cuts in early 2025 led by the US government and other development partners have sent shockwaves through the global health community, leaving low- and middle-income countries to grapple with the consequences. These funding cuts occurred against a backdrop of stagnating public health expenditures, crippling debt servicing, and slowed economic growth following the COVID-19 pandemic [3], alongside stagnating progress in reducing maternal and neonatal mortality [4]. In this context, unprecedented reductions in donor support threaten to roll back years of progress in maternal and neonatal health. Rasanathan et al. [3] urged global partners to support transition planning and to rethink global financing architectures in ways that promote equity, accountability, and national ownership, echoing calls, such as the Lusaka Agenda to enhance the effectiveness of GHIs.

Established in 2015, the GFF was designed to drive greater investment in maternal, newborn, and child health [5]. By leveraging targeted trust fund contributions, the GFF seeks to stimulate public financing, to ensure sustainable and long-term support [6]. Implementation in beneficiary countries has been guided by various key documents, including the Investment Case (IC) and Project Appraisal Document (PAD) [7].

The Countdown to the 2030 Health Policy and Systems Group brought together partners across countries in Sub-Saharan Africa to develop the papers in this *Global Health Action* Special Issue [2]. There are nine research articles: four country case studies that seek to understand the development process of GFF country documents [8–11], four multi-country studies that seek to assess the content of GFF documents and the extent to which they respond to country priorities [12–15], and one overarching paper [7]. Together they contribute to understanding the design, implementation (including stakeholder engagement) and challenges of the GFF in supporting reproductive, maternal, newborn, child, and adolescent health (RMNCAH) in 28 countries.

In this Commentary article we reflect on the content analysis of GFF country-planning documents and the extent to which they respond to national country priorities, followed by examination of the processes through which these documents were developed. In light of the recent global financing shifts, we focus on the Lusaka Agenda Shift 2 on sustainable financing [1], and discuss GFF’s progress in strengthening sustainable country-financed health systems.

## Content analysis: does the GFF address country priorities?

Four papers examined the GFF documents (IC and PAD) for content related to maternal health, neonatal health, stillbirths, adolescent health, community health, and quality [8–11]. The authors applied an analysis framework highlighting the mindset (content), measures (indicators), and money (investments) of the relevant themes [7]. This framework was a useful analytical approach, aligning with the Lusaka Agenda’s Shift 4 [1], which emphasizes making GHIs more responsive to the priorities of countries and communities.

The first missed opportunity identified was the content imbalance between maternal and newborn health interventions. There was limited attention to postnatal, small and sick newborn care and stillbirths in early GFF documents [15]. This is significant, as nearly half of the annual maternal, perinatal, child, and adolescent deaths occur globally among stillborn (1.9 million) and neonates [15,16]. We argue that stillbirth – a key red flag for poor intrapartum care – has received limited investment and is not a key indicator of health system performance, despite the significant mortality burden [15]. Furthermore, stillbirths are poorly reported and not prioritized for review, resulting in critical gaps in policy attention [17] which ultimately contribute to slow progress in reducing stillbirths. During the development of GFF ICs and PADs, critical appraisal of evidence on disease burden and gaps is essential to guide prioritization and investment decisions. Strengthening perinatal surveillance and response systems to reduce stillbirths and improve maternal and newborn outcomes across countries in Sub-Saharan Africa should be a key priority for GFF, offering clear returns on investment with lives saved.

The content analysis on quality of care for maternal and neonatal health revealed that most of the focus was on the provision of maternal health care, neglecting the experience of care for women and newborns [12]. Maternal health programs have historically prioritized clinical interventions over patient-centred approaches, leaving respectful maternity care underemphasised [18]. This content analysis would have been strengthened by placing more emphasis on the essential enablers of quality care – such as the availability of equipment, trained personnel, blood supplies, and other critical infrastructure. Blood transfusion, for example, plays an important role in addressing maternal health complications, particularly obstetric haemorrhage, which is a leading cause of maternal mortality.

The content analysis of community health revealed that the ICs reflected the countries’ commitment to community health, a critical tenet of PHC [13]. However, most community health indicators focus on Community Health Worker (CHW) deliverables and training, with limited attention paid to addressing governance and funding challenges that lead to demotivation and poor performance. There is a missed opportunity here to invest in broader community health programs and long-term financing of CHW programs. GFF has an opportunity to support the meaningful and sustainable engagement of CHWs and communities.

## The GFF policy processes: achieving strategic and operational cohesion

National alignment under the GFF was often achieved through the use of existing health agendas and systems, with ICs generally aligned to national plans [7]. However, World Bank planning processes influenced the development of the PADs [7]. Unclear communication regarding financing created confusion among stakeholders about disbursement modalities and accountability mechanisms. Some civil society organizations (CSOs) and local governments were excluded from fiscal planning, weakening transparency, equity, multi-sectoral commitments, implementation practices, and public oversight. While the GFF has made notable efforts to integrate its financing into national systems, inconsistencies between program design and implementation remain. Moreover, the tendency to prioritize short-term, visible results over long-term system investments undermines sustainability. This runs counter to the Lusaka Agenda’s Shift 1 [1], which emphasizes the need to strengthen PHC as the core of health systems and build lasting institutional capacity.

In Uganda the IC was closely tied to the country’s *Promise Renewed* Strategy and coordinated by an existing Technical Working Group in the Ministry of Health, thereby avoiding parallel structures [8]. However, the PAD development followed a separate track, led by the Ministry of Finance, Planning, and Economic Development and World Bank teams, to secure parliamentary approval for loans linked to the GFF. While the Parliamentary process helped strengthen ownership and policy fit, there was a missed opportunity to better coordinate between health and finance processes and stakeholders.

In Tanzania, strategic alignment was relatively strong due to the integration of GFF-supported processes into national frameworks, such as the Health Sector Strategic Plans IV and V. However, operational coherence suffered during the development of PAD 1, and this was primarily due to the early dominance of World Bank consultants and the limited inclusion of key national actors [11]. This led to fragmented coordination between IC and PAD, exacerbated by staff turnover and gaps in institutional memory. The missed opportunity was the failure to build institutional capacity and national ownership from the start. Although PAD 2 has been more inclusive, gaps remain in community engagement and stakeholder understanding of GFF mechanisms. Furthermore, in Burkina Faso, the PAD was approved before the IC, which further complicated financial planning and system integration [9].

The Lusaka Agenda Shifts 1 and 2 emphasize the need for inclusive, whole-of-society approaches that address the social and structural determinants of health [1]. Yet, in many countries, limited multisectoral collaboration and exclusion of marginalized groups, youth, and disability advocates hindered the equitable delivery of RMNCAH services [7]. A missed opportunity for the GFF at inception was the failure to enable the meaningful participation of diverse stakeholders. For the Lusaka Agenda to be realized, the GFF must move beyond rhetorical inclusion and institutionalize mechanisms that ensure the early and sustained participation of diverse stakeholders. This also means resourcing CSOs and community groups to participate effectively and proactively to address invisible power dynamics that systematically exclude vulnerable populations from policy processes.

To strengthen its alignment with national processes, the GFF could *borrow a leaf* from the Global Fund to Fight AIDS, Tuberculosis and Malaria (the Global Fund), where priorities are defined by the Country Coordinating Mechanism (CCM), a multi-stakeholder platform for decision-making and grant oversight [19]. The Global Fund mandates that the CCM includes representatives not only from government agencies and technical partners, but also from CSOs, affected communities (e.g. people living with HIV), and private sector actors. This ensures that there are diverse perspectives on funding allocation and program implementation. To promote inclusivity, the Global Fund has developed a Community Engagement Strategic Initiative that provides resources and technical assistance to strengthen CSO participation in grant processes [20]. Given the GFF’s focus, community engagement should place emphasis on gender equity.

## Does GFF catalyse sustainable, country-financed health systems?

The evidence collated across GFF country experiences presents a mixed picture of progress and reversal once funding ends. We argue that the GFF has made limited progress in catalysing sustainable domestic financing [21]. Below, we highlight three core aspects: a) the influence on domestic resource mobilization (DRM), b) combining debt commitments and grants, and c) the use of results-based financing.

### Contributions to increased domestic mobilisation

Despite the GFF’s emphasis on DRM, evidence of sustained financial planning and increased domestic investment in RMNCAH remains limited. While several countries have successfully integrated GFF funds into their national budgets, few have demonstrated genuine progress in DRM. Most PADs remained dominated by World Bank loan structures [7], and in contexts such as Burkina Faso and Uganda, domestic allocations for RMNCAH remained unchanged or minimal. For example, in Burkina Faso, the GFF’s financial contribution (US$20 million) primarily covered government arrears related to the free maternal and child healthcare policy rather than stimulating fresh domestic investment [9]. Instead of supplementing government spending, this financing substituted existing obligations, failing to incentivize greater fiscal commitment or public financial management reforms.

Despite developing three budget scenarios in the Uganda IC, there is no documented increase in domestic health financing attributable to the GFF process [8]. In Kenya the IC and PAD, indicated that GFF support for the procurement of family planning commodities would decline as domestic investment for family planning commodities increased. However, between 2016 and 2018/19, the government’s contribution decreased, increasing commodity insecurity [22].

Across the board, independent monitoring of DRM efforts was lacking, and political constraints limited the state’s capacity to pursue new tax reforms or long-term financing strategies [7]. When involved, the CSOs and private actors typically entered discussions late, diminishing their ability to advocate for fiscal accountability or sustainability. Ministries of Finance were not consistently engaged in budget planning, and transition strategies were often vague.

The GFF’s limited impact on DRM reflects a broader challenge in global health financing – the need for clear transition pathways and stronger national ownership of fiscal strategies. The Lusaka Agenda advocates for a reduction in dependence on donor funds and a shift towards sustainable financing models. However, the heavy reliance on loan-based funding and the absence of robust domestic financing strategies reinforces external dependency rather than fostering long-term fiscal autonomy. GFF could explore lessons from multilaterals, such as United Nations Population Fund, which require governments to pay for an agreed proportion of commodities to measure genuine commitment [23].

### A trojan horse? Debt pressures and the unintended impacts of GFF

Low- and middle-income countries face severe fiscal constraints from rising debt obligations, leaving limited space for additional health investments [3]. Many governments rely on external loans, including those facilitated by the GFF, and struggle to manage debt repayments and meet urgent health system needs. Evidence from the papers in this Special Issue underscores several important points. The GFF financing model often requires countries to accept both loans and grants. This means that governments need to take on additional debt, even for recurrent costs such as medicines, CHW stipends, and supervision. This approach undermines the long-term sustainability of GFF resources because servicing debt for routine health expenditures is fiscally imprudent.

Tanzania’s experience highlights the complexity of debt-driven health financing. GFF-supported investments were integrated into national systems through health basket funds and a Sector-Wide Approach, commonly called a SWAp, where national governments lead partnerships with donors and development partners. However, the core of the investment was through International Development Association loans, often called IDA or low-interest loans. Debt service remains a major constraint in fiscal planning across countries. The World Bank’s role in structuring financial terms and performance metrics skewed priorities towards short-term visible results over a deeper fiscal transformation. Country-led negotiations should be amplified to offset debt, as has been the case during the *Highly Indebted Poor Countries’ Initiative* since the late 1990s [24].

### Scaling up results-based financing (RBF) and fiscal sustainability

This Special Issue shows that RBF mechanisms, popularized by the World Bank, were widely employed in GFF-supported programs. In Mozambique, Uganda, Burkina Faso, and other countries, GFF funds were disbursed as performance-based grants to health facilities [7]. In Mozambique, stakeholders raised concerns about the lack of transparency in adopting the RBF approach [10]. However, RBF programs have shown mixed results in terms of strengthening health systems [25,26]. Challenges faced include sustainability, neglect of non-reimbursed services, equity, setup of parallel systems, and administrative complexities [27].

The Uganda Reproductive, Maternal, and Child Health Improvement Project attempted to scale up RBF from a scheme to a system reform in over 90% of the districts [28]. At the end of the project, RBF provided 69% discretionary income across government-owned facilities [29]. The project allowed flexibility in staff recruitment and medical procurement outside the National Medical Stores. These efforts attempted to offset the rigidities of the public finance management system, however, the changes could not be sustained beyond the project. For example, staff motivation, facility funding, service utilization, quality outcomes, and reporting quality declined immediately after the program ended, illustrating the volatility of donor-dependent RBF models [30]. Uganda is now exploring ways to mainstream RBF within national public finance management systems. However, many aspects of existing RBF structures (such as regular performance verifications and bonus payments) are not scaled up, resulting in lost investments [31].

From the outset, GHIs should align with existing public finance management frameworks to ensure continuity beyond the donor cycles; otherwise opportunities for sustained systems reform are missed. They should complement, rather than distort, broader health financing strategies, balancing incentives with holistic service delivery and equitable resource allocation.

## Conclusion

While GHIs are well-intentioned, they frequently fall short of delivering the systemic change envisioned in the Lusaka Agenda’s five strategic shifts. The GFF was established to catalyse domestic investments in health systems, aligning with the Lusaka Agenda’s call for a structured transition away from reliance on GHIs. This shift is becoming increasingly urgent, as reductions in global health aid and economic pressures strain national budgets.

This Commentary highlights how the papers in this *Global Health Action* Special Issue show missed opportunities where the GFF did not align with the Lusaka Agenda. The influence of external actors in operational planning misaligns with the Lusaka Agenda’s emphasis on the coherence between global initiatives and national systems. Parallel planning structures, operational inconsistencies, and unclear funding pathways hamper effective implementation. The inconsistencies between ICs and PADs reflect a missed opportunity for the GFF to demonstrate how global initiatives can fully align with and strengthen national planning and budgeting processes

To better align with the Lusaka Agenda vision, future global health financing efforts, including the GFF, must:

- Critically appraise evidence on disease burden and consistent gaps to inform prioritization and align GHI investment decisions.
- Effectively and meaningfully engage with stakeholders, particularly CSOs and vulnerable groups, in prioritizing investment decisions.
- Engage Ministries of Finance beyond loan negotiations to ensure that DRM strategies are embedded in broader fiscal planning.
- Align financing mechanisms with national public finance management systems to prevent reliance on donor-driven performance incentives, which may not be scalable in the long term.

Without addressing the missed opportunities, global financing mechanisms risk perpetuating short-term gains without addressing the systemic fiscal and operational challenges that hinder long-term health-system resilience.

## Data Availability

No new datasets were generated or analyzed in this study. Data sharing was not applicable to this study.
